# Safety and efficacy of indocyanine green near-infrared fluorescent imaging-guided lymph nodes dissection during radical gastrectomy for gastric cancer: A systematic review and meta-analysis

**DOI:** 10.3389/fonc.2022.917541

**Published:** 2022-08-16

**Authors:** Chun Deng, Zhenyu Zhang, Hengduo Qi, Zhi Guo, Yang Liu, Haimin Xiao, Xiaojun Li

**Affiliations:** ^1^ Department of Gastrointestinal Surgery, the Second People’s Hospital of Yibin, Yibin City, China; ^2^ Department of General Surgery, Shaanxi Provincial People’s Hospital, Xi’an City, China

**Keywords:** indocyanine green, laparoscopic surgery, lymph node, gastric cancer, robotic gastrectomy, meta-analysis

## Abstract

**Background:**

Indocyanine green (ICG) fluorescence imaging has been a new surgical navigation technique for gastric cancer. However, its clinical value should still be evaluated further. In this meta-analysis, we investigated the safety and efficacy of ICG near-infrared fluorescent imaging-guided lymph nodes (LNs) dissection during radical gastrectomy.

**Methods:**

Studies comparing ICG fluorescence imaging with standard care in patients with gastric cancer were systematically searched from PubMed, Embase, Web of Science, and Cochrane Library through August 2021. The current meta-analysis was performed according to the preferred reporting items for systematic review and meta-analysis guidelines. A pooled analysis was performed for the available data regarding the number of LNs dissection, the number of metastatic LNs dissection, other operative outcomes, and postoperative complications. R software version 4.2.0 and Stata 16.0 software were used for the present meta-analysis.

**Results:**

This analysis included 12 studies with a total of 1365 gastric cancer patients (569 in the ICG group and 796 in the non-ICG group). The number of retrieved LNs in the ICG group was significantly higher (weighted mean difference [WMD]=7.67, 95% confidence intervals [CI]: 4.73 to 10.62, P<0.05) compared to the non-ICG group with moderate heterogeneity (P<0.001, I^2 =^ 70%). The number of metastatic LNs, operative time, and postoperative complications were all comparable and without significant heterogeneity. Additionally, ICG near-infrared fluorescent imaging was associated with reduced intraoperative blood loss (WMD=-10.28, 95% CI: -15.22 to -5.35, P<0.05) with low heterogeneity (P=0.07, I2 = 43%).

**Conclusions:**

ICG near-infrared fluorescent imaging-guided lymphadenectomy was considered to be safe and effective in gastrectomy. ICG was used to increase the number of LNs harvested while reducing intraoperative blood loss without increasing operative time or postoperative complications.

**Systematic Review Registration:**

https://www.crd.york.ac.uk/PROSPERO/, identifier CRD42021291863.

## Introduction

Gastric cancer is the fifth most prevalent malignant tumor in the world and the fourth leading cause of cancer mortality (1). Lymph node (LN) metastasis is a major risk factor for recurrence and metastasis of gastric cancer (2–5), and LN dissection is a standard procedure of radical gastrectomy. Owing to the intricacy of the anatomy and the rich blood supply, adequate LN dissection is a substantial challenge during radical gastrectomy (6–9).

Indocyanine green (ICG), an FDA-approved dye for *in vivo* use, offers superior assessment of blood and lymphatic vessels (10). With the simple process of ICG fluorescence imaging, ICG fluorescence imaging is a hot spot revealing its superiority in a variety of oncological surgery (11–17). In a radical gastrectomy, it enables the surgeon to accurately observe the perigastric LNs at a closer distance and under closer physiological conditions, it can also locate the LNs precisely to guide the surgeon during the operation in real-time (18–20). However, since the application of ICG in lymphadenectomy for patients with gastric cancer is still in the preliminary stages, its safety and efficacy remain unclear.

To date, we have only found one meta-analysis evaluating the safety and efficacy of ICG near-infrared fluorescent imaging-guided radical gastrectomy (21), it included six articles, two of which were published in Chinese. However, this meta-analysis failed to include eight very important studies that had already been published (22–29). Moreover, no subgroup analysis was performed, and two of them were published in Chinese domestic journals with a relatively low quality of evidence rather than in international journals. Through the analysis of twelve English literature, we performed an updated systematic review and meta-analysis to provide more comprehensive and reliable evidence, with the primary outcome being the total number of LNs retrieved and the secondary outcomes being the number of metastatic LNs, operative time, intraoperative blood loss, and postoperative complications.

## Materials and methods

A systematic literature review and meta-analysis were performed according to the PRISMA guidelines (30). The protocol was registered in the PROSPERO register before starting this systematic review and meta-analysis (CRD42021291863).

### Study objective

In this meta-analysis, the primary endpoint was the total number of retrieved LNs, and the secondary endpoints were the number of metastatic LNs, operative time, intraoperative blood loss, and postoperative complications. The effectiveness of ICG near-infrared fluorescent imaging-guided LNs dissection was assessed by the total number of retrieved LNs and the number of metastatic LNs, while safety was assessed by operative time, intraoperative blood loss, and postoperative complications.

### Search strategy

Through November 2021, all relevant studies from Embase, Web of Science, PubMed, and the Cochrane Library were systematically reviewed. The search strategy contained two core components, which were linked using the AND operator: 1) stomach neoplasms (e.g., neoplasm, stomach, stomach neoplasm, neoplasms, stomach, gastric neoplasms, gastric neoplasm, neoplasm, gastric, neoplasms, gastric, stomach cancers, cancer of stomach, gastric cancer, cancer, gastric, cancers, gastric, gastric cancers, stomach cancer, cancers, stomach, cancer, stomach, cancer of the stomach, gastric cancer, familial diffuse), 2) indocyanine green (e.g., green, indocyanine, wofaverdin, vophaverdin, ujoveridin, vofaverdin, cardio-green, cardio green, cardiogreen. For each of the two core components, controlled vocabulary (i.e. Medical Subject Headings terms) and title/abstract were identified. The search was developed initially for PubMed and then adapted for each of the other three databases by mapping the search terms to additional controlled vocabulary and subject heading terminology. The search was carried out separately by two authors (DC and ZZ) with no language or date restrictions (Supporting References 1).

### Study selection

The studies that were included met the following criteria: (1) patients were diagnosed with gastric cancer; (2) the study included patients with and without ICG tracer-guided radical gastrectomy; (3) the article with the most complete data for studies with duplicated data; and (4) retrospective and prospective research as well as randomized controlled trials (RCTs).

The following were the criteria for exclusion: (1) case studies, reviews, comments, correspondence, and animal studies; (2) studies with insufficient data for analysis; and (3) repeated studies by the same author.

### Data extraction and study quality

For the eligible studies, two authors (DC and ZZ) independently extracted the data and any disagreements were resolved through quality control discussions with another author (HQ) whenever necessary. The following information was recorded: the first author, publication year, nation, study design, time period of this study, number of patients in ICG group and non-ICG group, operative approach, the number of retrieved LNs, the number of metastatic LNs, operative time, intraoperative blood loss, and postoperative complications.

The RCT’s quality was determined using the Cochrane risk assessment tool (31). Studies with a score of 4 points were considered high-quality studies, with a maximum score of 6 points. The quality of nonrandomized controlled studies was defined by Newcastle–Ottawa Quality Assessment Scale (NOS) (32). Studies with scores of 6 points or higher were considered high-quality studies. Two researchers independently evaluated the quality of each study.

### Statistical analysis

The odds ratio (OR) and weighted mean difference (WMD) with their corresponding 95% confidence intervals (CI) were used to analyze dichotomous and continuous variables. For studies that only offered median and range, data were converted to mean and standard deviation (SD) using the method described by Wan et al (33). The Chi-square and I^2^ tests were used to assess statistical heterogeneity. P<0.10 was used as the significance level for heterogeneity. Heterogeneity was deemed acceptable when P>0.10 and I^2^<50%. It was then tested using a fixed-effects model. A random-effects model was applied if I2>50%. I^2^<50% was regarded to represent low heterogeneity, while 50% to 75% and≥75% indicated moderate and high heterogeneity, respectively. For the source of heterogeneity, a sensitivity analysis of each study and subgroup analysis were used for secondary analysis. The Egger’s and Begg’s tests were used to evaluate publication bias. P<0.05 was regarded as significant. All of the statistical analyses were performed by R software version 4.2.0 (R Foundation, Vienna, Austria) and Stata 16.0 software (StataCorp, College Station, TX, USA).

## Results

### Characteristics of studies

**Figure 1:**
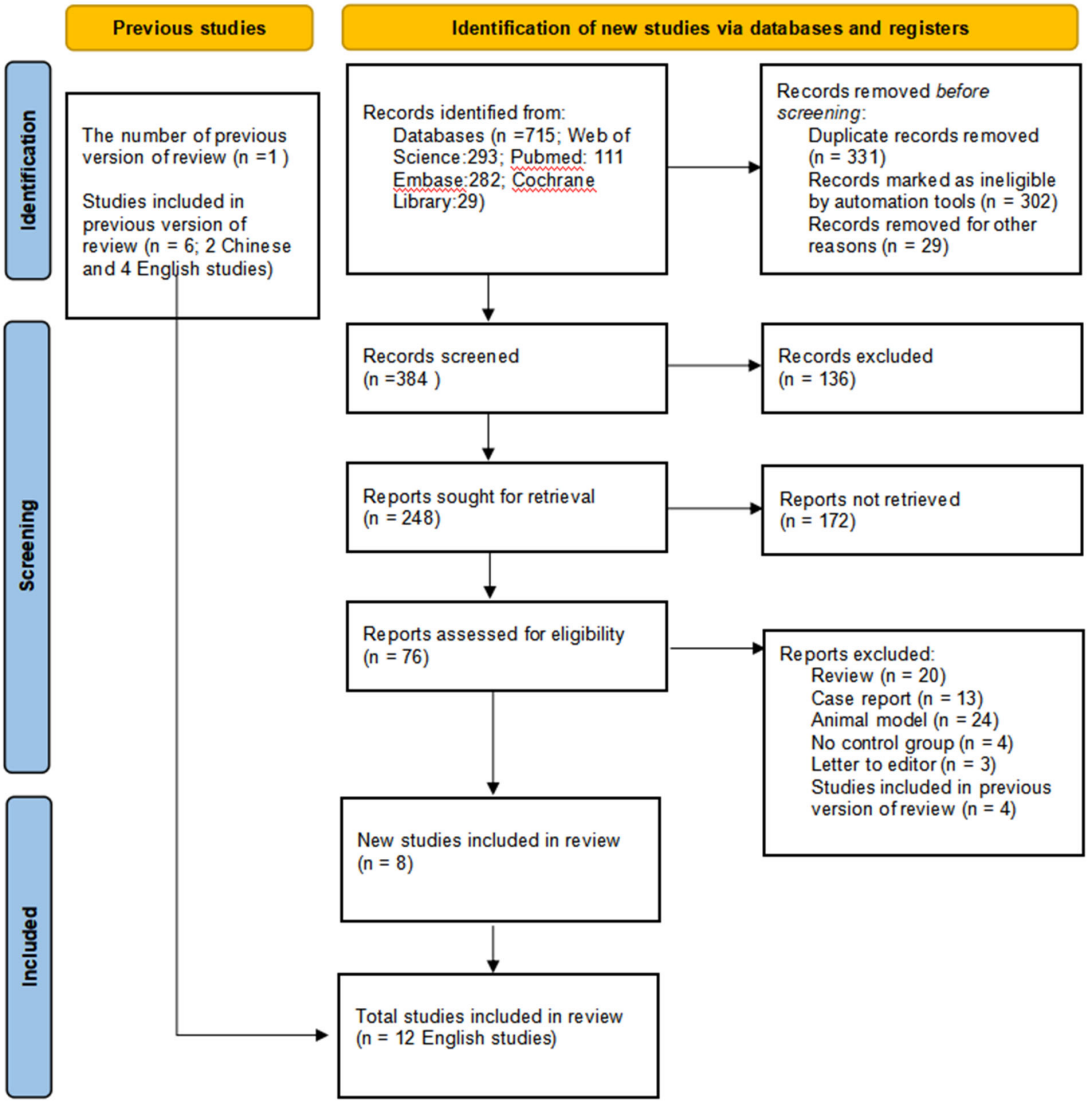
PRISMA flowchart of literature search and selection process. PRISMA, Preferred Reporting Items for Systematic Reviews and Meta-Analysis.

A total of 12 English papers were included in the meta-analysis after a literature search and selection based on the inclusion criteria (15, 22–29, 34–36). The details of the selection procedures were shown in accordance with the PRISMA flowchart (**Figure 1**). **Table 1** summarizes the general information from the studies that were included. A total of 1365 patients with stomach cancer were included in this meta-analysis (569 in the ICG group and 796 in the Non-ICG group). All of the research were published between 2017 and 2021 and came from three different nations (Korea, Italy, and China). The sample size ranged from 20 to 290 patients.

**Table 1:** Study characteristics.

First author	Year	Country	Studyinterval	Studydesign	Sample size(I:C)	Operationmethod	The way of ICG injection	The time of injection	NOS	Cochrancescore
Liu (22)	2020	China	2017-2019	S; R	61:75	laparoscopic gastrectomy	Endoscopy(submucosa)	Intraoperatively	7	–
Tian (29)	2021	China	2019-2020	S; R	27:32	robotic gastrectomy	Endoscopy(submucosally)	1 day before surgery	8	–
Lan (36)	2017	China	2011-2016	S; R	14:65	robotic gastrectomy	Endoscopy (submucosal) Chiba needle(subserosal)	Intraoperative or1 day before surgry	7	–
Ma (28)	2020	China	2018-2019	S; R	31:34	laparoscopic gastrectomy	Endoscopy(unclear)	within 12 h before surgery	8	–
Roh (27)	2020	Korea	2014-2017	S; R	98:192	laparoscopic or robotic gastrectomy	Endoscopy(submucosa)	1 day before surgery	8	–
Lee (26)	2021	Korea	2013-2018	S; R	74:94	laparoscopic or robotic gastrectomy	Endoscopy(submucosal)	1 day before surgery	8	–
Park (25)	2020	Korea	2017-2018	S; R	20:60	laparoscopic gastrectomy	Endoscopy(submucosal)	Intraoperatively	8	–
Cianch (35)	2019	Italy	2014-2018	S; R	37:37	robotic gastrectomy	Endoscopy(submucosa)	1 day before surgery	7	–
Kwon (34)	2018	Korea	2012-2014	S; P	40:40	robotic gastrectomy	Endoscopy(submucosal)	1 day before surgery	8	–
Romanzi (24)	2020	Italy	2017-2019	S; P	10:10	Robotic Gastrectomy	Endoscopy(submucosal)	18h before surgery	7	–
Lu (23)	2021	China	2015-2019	S; R	28:28	laparoscopic gastrectomy	Endoscopy(unclear)	Intraoperatively	8	–
Chen (15)	2020	China	2018-2019	RCT	129:129	laparoscopic gastrectomy	Endoscopy(submucosa)	1 day before surgery	–	5

ICG, indocyanine green; I ICG, S, single centre; R, retrospective study; P, prospective; NOS, Newcastle–Ottawa.

### Characteristics of ICG injection

The characteristics of ICG injection are displayed in **Table 2**. Most studies used endoscopy for submucosal injection, which was performed intraoperatively or one day before surgery. There were no ICG-related complications reported in any of these studies.

**Table 2:** Characteristics of ICG injection.

Study	The way of ICG injection	The time of ICG injection	ICG injection site	ICG injection concentration	ICG injection dose	Complications related to ICG
Liu 2020 (22)	Endoscopy	Intraoperative	Four points (proximal, distal, and bilateral to the tumor region), submucosal injection.	0.625 mg/mL	0.5 ml for each point, 2 ml in total.	None
Tian 2021 (29)	Endoscopy	1 day before surgery	4 points around the primary tumor, submucosal injection.	Not indicated	0.5 ml for each point, 2 ml in total.	None
Lan 2017 (36)	Endoscopy or Chiba needle (18 gauge)	Intraoperative or1 day before surgry	4 points around the primary tumor, Chiba needle (18 gauge) for subserosal injection, endoscopic injection for submucosal injection.	2.5 mg/mL	0.6 ml for each point, 2.4 ml in total.	None
Ma 2020 (28)	Endoscopy	within 12 h before surgery	3 points in lesser curvature, 4 points in greater curvature, Spaced from adoral to aboral sites as equally as possible.	1.25 mg/ml	0.5ml each point, 3.5 ml in total.	None
Roh 2020 (27)	Endoscopy	1 day before surgery	4 points around the ESD scar, submucosal injection.	1.25 mg/ml→0.625 mg/ml	0.6 ml for each point, 2.4 ml in total.	None
Lee 2021 (26)	Endoscopy	1 day before surgery	4 points around the primary tumor, submucosal injection.	1.25 mg/ml→0.625 mg/ml	0.6 ml for each point, 2.4 ml in total.	None
Park 2020 (25)	injection needle (23-gauge)	Intraoperative	five locations (on the lesser curva- ture side—low body and antrum; on the greater curvature side—mid body, low body and antrum), submucosal injection.	0.1 mg/mL	1 ml for each point, 5 ml in total.	None
Cianch 2019 (35)	Endoscopy	1 day before surgery	4 points around the primary tumor, submucosal injection.	1.25 mg/ml	0.5 ml for each point, 2 ml in total.	None
Kwon 2018 (34)	Endoscopy	1 day before surgery	4 points around the primary tumor, submucosal injection.	1.25 mg/mL	0.6 ml for each point, 2.4 ml in total.	None
Romanzi 2020 (24)	Endoscopy	18h before surgery	4 points around the primary tumor, submucosal injection.	1.25mg/ml	0.6 ml for each point, 2.4 ml in total.	None
Lu 2021 (23)	Endoscopy	Intraoperative	proximal and distal submucosa of the tumor.	2.5mg/ml	0.5ml at a time, didn’t reported the total dose	None
Chen 2020 (15)	Endoscopy	1 day before surgery	4 points around the primary tumor, submucosal injection.	1.25 mg/mL	0.5 ml for each point, 2 ml in total.	None

Complications related to ICG: ICG-induced nausea, fever, allergy and shock symptoms.

### The main objective

#### The number of retrieved LNs

**Figure 2:**
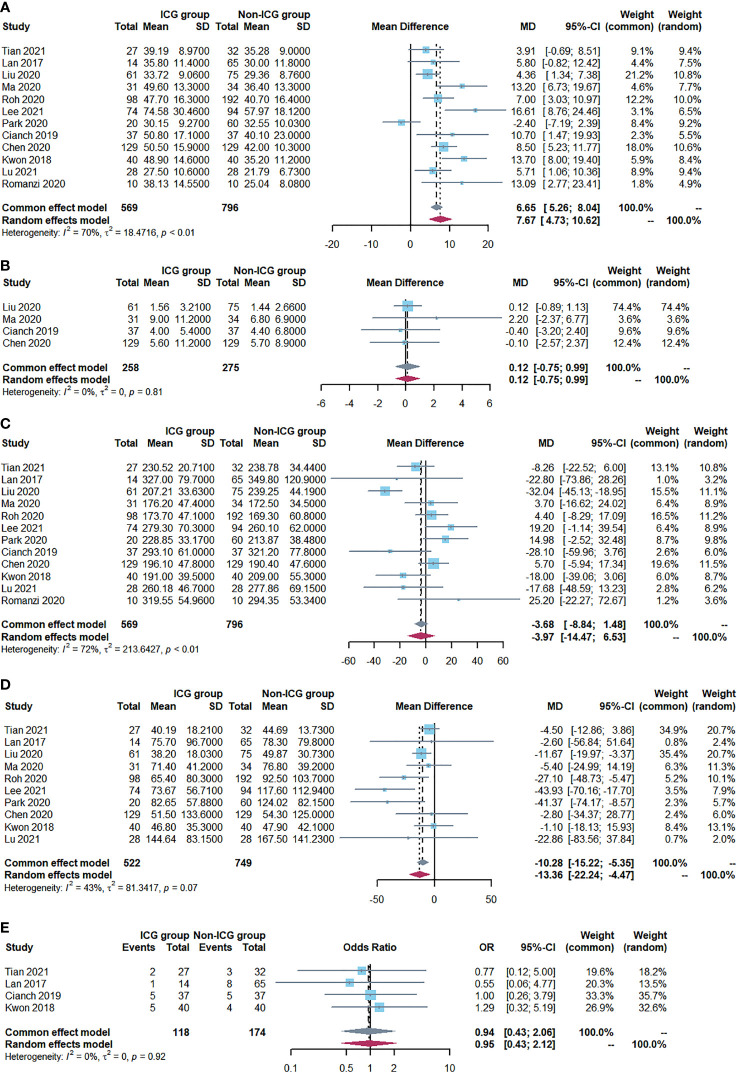
Forest plots showing outcome assessment including **(A)** the number of retrieved lymph nodes, **(B)** the number of metastatic lymph nodes, **(C)** operative time, **(D)** Intraoperative blood loss and **(E)** postoperative complications.

The primary outcome of this study was to assess ICG near-infrared fluorescent imaging on the number of retrieved LNs after radical gastrectomy. Ultimately, 12 studies (1365 patients) were included in our meta-analysis. Among these patients, the number of retrieved LNs in the ICG group was higher compared to the non-ICG group. This meta-analysis found that ICG fluorescent imaging had a positive effect on increasing the number of retrieved LNs (WMD=7.67, 95% CI: 4.73 to 10.62, P<0.05) with moderate heterogeneity (P<0.001, I^2 =^ 70%) as shown in **Figure 2A**.

### The secondary objectives

#### The number of metastatic LNs

A fixed-effects model was adopted since there was no evidence of heterogeneity (P=0.81; I2 = 0%). The analysis included four studies (534 patients) (**Figure 2B**). In both groups, there was no significant difference in the number of metastatic LNs (WMD=0.12, 95% CI: -0.75 to 0.99, P=0.79).

### Operative time

Twelve studies reported the operative time. Meta-analysis showed no difference in operative time between the two groups (WMD=-3.97, 95% CI: -14.47 to 6.53, P=0.46) with moderate heterogeneity (P<0.001, I^2 =^ 72%), as shown in **Figure 2C**.

### Intraoperative blood loss

Ten studies reported intraoperative blood loss, and all of the studies demonstrated a statistically significant reduction in the ICG group compared with the Non-ICG group. The meta-analysis demonstrated that the patients in the ICG group had a mean reduction of 10.28 ml in intraoperative blood loss compared to the patients in the non-ICG group (WMD=-10.28, 95% CI: -15.22 to -5.35, P<0.05) with low heterogeneity (P=0.07, I^2^ = 43%) as shown in **Figure 2D**.

### Postoperative complications

The postoperative complication rate was reported in nine studies (975 patients). No significant difference was observed between the ICG group (22.2%; n =98/441) and the non-ICG group (25.7%; n =137/534) regarding this outcome (OR 0.94, 95% CI 0.43 to 2.06, P=0.88) with no heterogeneity (P=0.92, I2 = 0%) (**Figure 2E**).

### Sensitivity analysis

To assess the impact of single studies and analyze the effects of heterogeneity on the pooled WMDs of the number of retrieved LNs, we performed sensitivity analysis by sequentially removing one study from the overall pooled analysis. When we excluded any study, the pooled WMDs and their corresponding 95% CIs were similar, according to the results of this sensitivity analysis. Hence, our findings were relatively consistent and reliable (Supporting References 2).

### Subgroup analysis

Subgroup analysis was used to explore the heterogeneity (**Table 3** and Supporting References 3). The following parameters of each study were included: operation type (robotic surgery versus laparoscopic surgery versus laparoscopic or robotic surgery), nation (China versus Korea versus Italy), and study design (retrospective study versus RCT versus prospective study). Subgroup analysis based on “operation type” suggested that ICG near-infrared fluorescent imaging had a positive effect on increasing the number of retrieved LNs in all kinds of operations (robotic surgery: WMD=8.80, 95% CI: 4.37 to 13.23, P<0.05; laparoscopic surgery: WMD=5.69, 95% CI: 1.03 to 10.35, P<0.05; laparoscopic or robotic surgery: WMD=11.18, 95% CI: 1.85 to 20.52, P<0.05). Inter-study heterogeneity was high and significant in the other three groups (I^2 =^ 53%, 80%, and 78%, respectively). In terms of “nation”, ICG tracer-guided lymphadenectomy was significantly associated with the increasing number of retrieved LNs in China (WMD=6.32, 95% CI: 4.61 to 8.02, P<0.05) with low heterogeneity (I^2 =^ 43%, P=0.12), Korea (WMD=8.41, 95% CI: 0.20 to 16.61, P<0.05) with significant heterogeneity (I^2 =^ 88%, P<0.01), and Italy (WMD=11.76, 95% CI: 4.88 to 18.64, P<0.05) with no significant heterogeneity (I2 = 0.0%, P=0.74). We found that ICG near-infrared fluorescent imaging could increase the number of LN harvested in the retrospective study (WMD=6.56, 95% CI: 3.14 to 9.99, P<0.05) with moderate heterogeneity (I^2 =^ 70%, P<0.01) and the prospective study (WMD=13.56, 95% CI: 8.57 to 18.55, P<0.05) with no significant heterogeneity (I^2 =^ 0.0%, P=0.92).

**Table 3:** Subgroup analysis of pooled WMD for the number of lymph node dissection.

Categories	No. of studies	No. of patients (I:C)	Meta-analysis				
			H (P, I^2^)	WMD	95%CI		P-value
Operation							
robotic surgery	5	312 (128:184)	P=0.07 I^2 =^ 53%	8.80	4.37 to 13.23	<0.001	
laparoscopic surgery	5	595 (269:326)	P<0.01 I^2 =^ 80%	5.69	1.03 to 10.35	0.012	
laparoscopic or robotic surgery	2	458 (172:286)	P=0.03 I^2 =^ 78%	11.18	1.85 to 20.52	0.019	
Nation							
China	6	653 (290:363)	P=0.12 I^2 =^ 43%	6.32	4.61 to 8.02	<0.001	
Korea	4	618 (232:386)	P<0.01 I^2 =^ 88%	8.41	0.20 to 16.61	0.036	
Italy	2	94 (47:47)	P=0.74 I^2 =^ 0%	11.76	4.88 to 18.64	0.001	
Study design							
retrospective study	9	1007 (390:617)	P<0.01 I^2 =^ 70%	6.56	3.14 to 9.99	<0.001	
RCT	1	258 (129:129)	–	8.50	5.23 to 11.77	<0.001	
prospective study	2	100 (50:50)	P=0.92 I^2 =^ 0%	13.56	8.57 to 18.55	<0.001	

NO. Number; I ICG group; C control group.

### Publication bias

**Figure 3:**
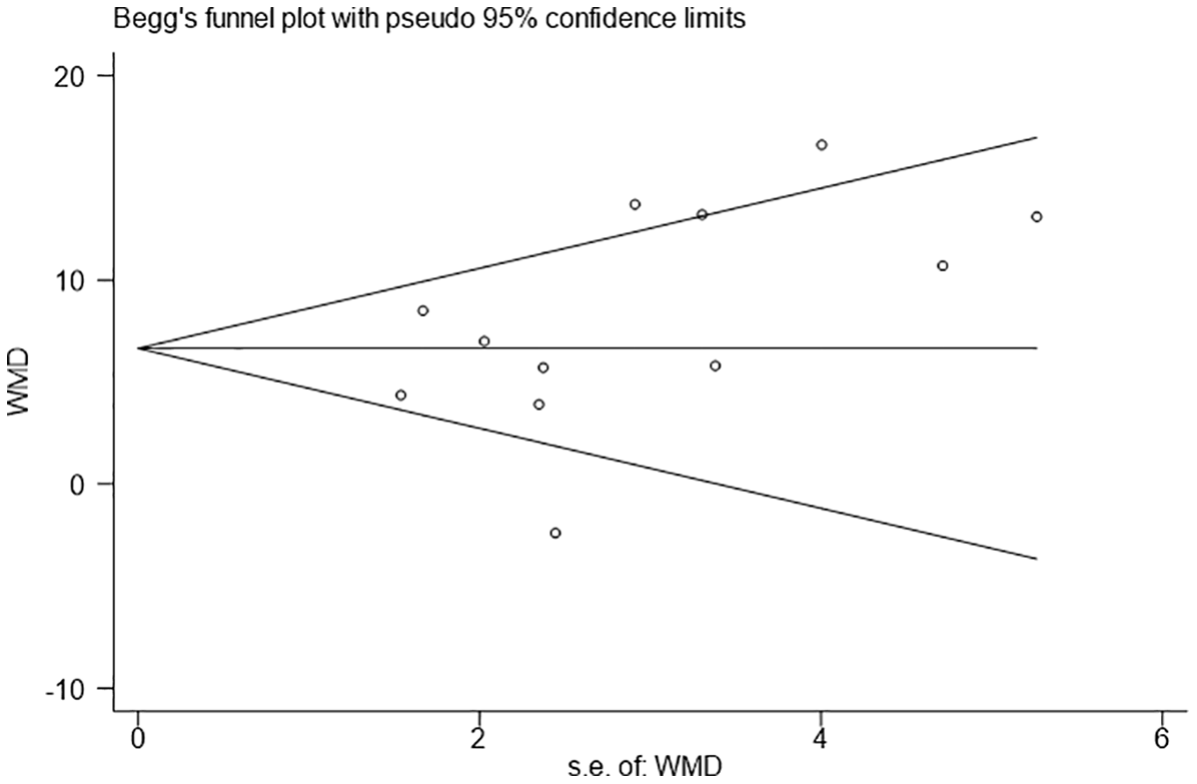
Funnel plots evaluating the relationship between the intraoperative use of Indocyanine green and the number of lymph node dissection.

The Egger’s and Begg’s tests were used to analyze the publication bias of our meta-analysis. There was no evidence of publication bias in the number of retrieved LNs (Egger P=0.141; Begg P=0.150) (**Figure 3**).

## Discussion

In recent years, ICG near-infrared fluorescent imaging-guided radical gastrectomy has become a new direction of exploration because it has better tissue penetration and better identification of LNs (37, 38). The results of our meta-analysis showed that ICG-guided radical gastrectomy can obtain more LNs and reduce intraoperative blood loss; it also has a similar operative time and postoperative complication rate as traditional radical gastrectomy, which indicates the safety and efficacy of ICG near-infrared fluorescent imaging.

ICG injection is essential for efficient intraoperative LNs imaging (39). Most studies (15, 22–24, 26–29, 34, 35) used endoscopy for submucosal injection, which was performed around the primary tumor one day before surgery. The results of these studies showed that it could increase the overall number of recovered LNs without increasing surgery-related complications. Some researchers hold a different opinion wherein intraoperative sub serosal injection by needle would be more convenient and can achieve comparable outcomes (25, 39) Compared with intraoperative sub serosal injection, preoperative submucosal injections increase patient discomfort and endoscopist workload, which greatly limits the application of this technique (39). In addition, the concentration of ICG solution was also important. Lee et al. (26) and Roh et al. (27) reduced the concentration of ICG from 1.25 mg/mL to 0.625 mg/mL in 2015 because fluorescence signals were too high to perform LN dissection.

The primary results in our analysis indicated that ICG near-infrared fluorescent imaging was significantly associated with a larger number of harvested LNs (WMD=7.59, 95% CI: 4.86 to 10.32, P<0.05). A previous meta-analysis also showed that the ICG group had a substantially larger number of dissected LNs (WMD= 7.69, 95% CI: 5.64 to 9.74, P<0.00001) (21). The higher number of harvested LNs in the ICG group might be attributed to two reasons: one, ICG near-infrared fluorescent imaging allowed surgeons to perform complete lymphadenectomy by distinguishing LNs from perigastric blood vessels, fat, pancreatic tissue, and other tissue (15, 40), and two, the increase in the number of retrieved LNs could be due to a more thorough retrieval of the LNs (27, 33). Even tiny LNs can be recognized due to the excellent sensitivity of ICG near-infrared fluorescence imaging (34, 41). Previous studies (21, 42–44) have shown that a larger number of LNs dissections was associated with better long-term survival of patients with gastric cancer. Therefore, ICG-guided radical gastrectomy may have a better prognosis. Certainly, it needs to be confirmed further by higher-quality evidence regarding long-term survival.

Theoretically, an increased total number of harvested LNs means a greater possibility of getting a higher number of metastatic LNs. Previous studies have shown that in colorectal cancer, ICG fluorescence imaging can guide the extent of LN dissection and obtain more positive LNs (45–47). However, our meta-analysis showed that the number of metastatic LNs in the ICG group was not meaningfully higher than the number in the non-ICG group. ICG is not a cancer-specific tracer and has a limited diagnostic value for metastatic LNs (48, 49), which is the biggest drawback of indocyanine green fluorescence imaging and the focus of the current research. Shao et al. (50) developed an RGD-modified distearyl acylphosphatidyl ethanolamine-polyethylene glycol micelle (DSPE-PEG-RGD) to encapsulate indocyanine green (ICG) and found that it had an improved accumulation in tumors and a longer circulation time. It is believed that better tracers will emerge in the future to guide the implementation of more precise radical gastrectomy.

Intraoperative blood loss, operative time, and postoperative complications were important factors in assessing the safety of surgery. Our analysis showed a significant reduction in intraoperative blood loss in the ICG group. Park et al. (25) found that near-infrared ICG fluorescence-guided lymphadenectomy can reduce the incidence of bleeding, especially for infrapyloric LNs dissection. This was most likely because surgeons found it easier to identify the avascular plane and distinguish the blood vessels from the surrounding lymphatic structures, lowering the danger of blood vessel injury (11, 25). However, it should be pointed out that the 10.28 ml reduction in bleeding volume is not clinically significant.

In our meta-analysis, we found no significant differences in operative time and postoperative complications between the ICG and non-ICG groups, which is similar to the meta-analysis of Yang et al. (21) ICG fluorescence imaging can assist in distinguishing lymphatic tissue, adipose tissue, and pancreatic tissue, which may not be discernible in some patients with advanced gastric cancer to the naked eye of the surgeon. Thus, it could assist surgeons to perform lymphadenectomy safely and effectively by preventing injuries (11, 19, 20).

Based on the above analysis results, the use of ICG during radical gastrectomy can extract more lymph nodes, minimize intraoperative blood loss, and have similar short-term effectiveness as a traditional radical gastrectomy. Since the use of ICG in lymph node dissection in patients with gastric cancer is in its preliminary stage, we found only one study reporting long-term outcomes in patients undergoing ICG-guided gastrectomy (23). The mean follow-up time in this study was 21.25 and 26.29 months in the ICG and non-ICG groups, respectively, and the long-term impact was similar in both groups (23). More and higher quality studies are needed to assess long-term survival, assess long-term outcomes, particularly recurrence-free survival and cumulative survival rates.

The following are some of the limitations of this study: (1) clinical heterogeneity: Due to the inherent weaknesses of retrospective studies, the homogeneity test for continuous variables revealed moderate heterogeneity; and (2) geographical disparity: The bulk of the papers considered were from East Asia, which has the greatest occurrence of stomach cancer, whereas gastric cancer is relatively uncommon in Western countries (51, 52). When evaluating the findings of our study, the foregoing limitations must be kept in mind.

## Conclusion

This meta-analysis showed that ICG near-infrared fluorescent imaging-guided gastrectomy is safe and effective. Nevertheless, high-quality studies with long-term follow-up are necessary to confirm this conclusion.

## Data availability statement

The original contributions presented in the study are included in the article/**Supplementary Material**, Further inquiries can be directed to the corresponding author.

## Author contributions

CD and XL made substantial contributions to conception and design for this work. CD, ZZ and HQ collected all the data. CD and ZG was the major contributor in writing the manuscript. YL and HX performed critical revision for this manuscript. All authors contributed to the article and approved the submitted version.

## Conflict of interest

The authors declare that the research was conducted in the absence of any commercial or financial relationships that could be construed as a potential conflict of interest.

## Publisher’s note

All claims expressed in this article are solely those of the authors and do not necessarily represent those of their affiliated organizations, or those of the publisher, the editors and the reviewers. Any product that may be evaluated in this article, or claim that may be made by its manufacturer, is not guaranteed or endorsed by the publisher.

## References

[1] Sung H Ferlay J Siegel RL Laversanne M Soerjomataram I Jemal A . Global cancer statistics 2020: GLOBOCAN estimates of incidence and mortality worldwide for 36 cancers in 185 countries. CA Cancer J Clin (2021) 71(3):209–49. doi: 10.3322/caac.21660 33538338

[2] Yanzhang W Guanghua L Zhihao Z Zhixiong W Zhao W . The risk of lymph node metastasis in gastric cancer conforming to indications of endoscopic resection and pylorus-preserving gastrectomy: A single-center retrospective study. BMC Cancer (2021) 21(1):1280. doi: 10.1186/s12885-021-09008-8 34837993 PMC8627613

[3] Izumi D Gao F Toden S Sonohara F Kanda M Ishimoto T . A genomewide transcriptomic approach identifies a novel gene expression signature for the detection of lymph node metastasis in patients with early stage gastric cancer. EBioMedicine (2019) 41:268–75. doi: 10.1016/j.ebiom.2019.01.057 PMC6441863 30772302

[4] Zhang N Deng J Wang W Sun Z Wang Z Xu H . Negative lymph node count as an independent prognostic factor in stage III patients after curative gastrectomy: A retrospective cohort study based on a multicenter database. Int J Surg (2020) 74:44–52. doi: 10.1016/j.ijsu.2019.12.018 31874262

[5] Bilici A Selcukbiricik F Seker M Oven BB Olmez OF Yildiz O . Prognostic significance of metastatic lymph node ratio in patients with pN3 gastric cancer who underwent curative gastrectomy. Oncol Res Treat (2019) 42(4):209–16. doi: 10.1159/000496746 30870846

[6] Japanese Gastric Cancer Association . Japanese Gastric cancer treatment guidelines 2018 (5th edition). Gastric Cancer (2021) 24(1):1–21. doi: 10.1007/s10120-020-01042-y 32060757 PMC7790804

[7] Wang FH Zhang XT Li YF Tang L Qu XJ Ying JE . The Chinese society of clinical oncology (CSCO): Clinical guidelines for the diagnosis and treatment of gastric cancer, 2021. Cancer Commun (Lond) (2021) 41(8):747–95. doi: 10.1002/cac2.12193 PMC8360643 34197702

[8] Guideline Committee of the Korean Gastric Cancer Association (KGCA) Development Working Group & Review Panel . Korean Practice guideline for gastric cancer 2018: An evidence-based, multi-disciplinary approach. J Gastric Cancer (2019) 19(1):1–48. doi: 10.5230/jgc.2019.19.e8 30944757 PMC6441770

[9] Ajani JA D’Amico TA Bentrem DJ National Comprehensive Cancer Network . NCCN clinical practice guidelines in oncology: Gastric Cancer.Version1.2019 (2019). Available at: https://www.nccn.org (Accessed March 14, 2019).

[10] Dijkstra BM Jeltema HJR Kruijff S Groen RJM . The application of fluorescence techniques in meningioma surgery-a review. Neurosurg Rev (2019) 42(4):799–809. doi: 10.1007/s10143-018-01062-4 30519770 PMC6821664

[11] Valente SA Al-Hilli Z Radford DM Yanda C Tu C Grobmyer SR . Near infrared fluorescent lymph node mapping with indocyanine green in breast cancer patients: A prospective trial. J Am Coll Surg (2019) 228(4):672–8. doi: 10.1016/j.jamcollsurg.2018.12.001 30582975

[12] Digesu CS Hachey KJ Gilmore DM Khullar OV Tsukada H Whang B . Long-term outcomes after near-infrared sentinel lymph node mapping in non-small cell lung cancer. J Thorac Cardiovasc Surg (2018) 155(3):1280–91. doi: 10.1016/j.jtcvs.2017.09.150 PMC5816699 29248292

[13] Dang JT Skulsky S Switzer N Tian C Shi X Skubleny D . Diagnostic evaluation of sentinel lymph node biopsy using indocyanine green and infrared or fluorescent imaging in gastric cancer: A systematic review and meta-analysis. Surg Endosc (2018) 32(6):2620–31. doi: 10.1007/s00464-018-6100-9 29484554

[14] Jeremiasse B van den Bosch CH Wijnen MWHA Terwisscha van Scheltinga CEJ Fiocco MF van der Steeg AFW . Systematic review and meta-analysis concerning near-infrared imaging with fluorescent agents to identify the sentinel lymph node in oncology patients. Eur J Surg Oncol (2020) 46(11):2011–22. doi: 10.1016/j.ejso.2020.07.012 32826112

[15] Chen QY Xie JW Zhong Q Wang JB Lin JX Lu J . Safety and efficacy of indocyanine green tracer-guided lymph node dissection during laparoscopic radical gastrectomy in patients with gastric cancer: A randomized clinical trial. JAMA Surg (2020) 155(4):300–11. doi: 10.1001/jamasurg.2019.6033 32101269

[16] Kitagawa H Namikawa T Iwabu J Fujisawa K Uemura S Tsuda S . Assessment of the blood supply using the indocyanine green fluorescence method and postoperative endoscopic evaluation of anastomosis of the gastric tube during esophagectomy. Surg Endosc (2018) 32(4):1749–54. doi: 10.1007/s00464-017-5857-6 28916846

[17] Ushimaru Y Omori T Fujiwara Y Yanagimoto Y Sugimura K Yamamoto K . The feasibility and safety of preoperative fluorescence marking with indocyanine green (ICG) in laparoscopic gastrectomy for gastric cancer. J Gastrointest Surg (2019) 23(3):468–76. doi: 10.1007/s11605-018-3900-0 30084063

[18] Herrera-Almario G Patane M Sarkaria I Strong VE . Initial report of near-infrared fluorescence imaging as an intraoperative adjunct for lymph node harvesting during robot-assisted laparoscopic gastrectomy. J Surg Oncol (2016) 113(7):768–70. doi: 10.1002/jso.24226 PMC4964277 27021142

[19] van der Poel HG Buckle T Brouwer OR Valdés Olmos RA van Leeuwen FW . Intraoperative laparoscopic fluorescence guidance to the sentinel lymph node in prostate cancer patients: clinical proof of concept of an integrated functional imaging approach using a multimodal tracer. Eur Urol (2011) 60(4):826–33. doi: 10.1016/j.eururo.2011.03.024 21458154

[20] Miyashiro I Kishi K Yano M Tanaka K Motoori M Ohue M . Laparoscopic detection of sentinel node in gastric cancer surgery by indocyanine green fluorescence imaging. Surg Endosc (2011) 25(5):1672–6. doi: 10.1007/s00464-010-1405-3 20976497

[21] Yang J Wang Z Dong K Zhang R Xiao K Shang L . Safety and efficacy of indocyanine green fluorescence imaging-guided radical gastrectomy: a systematic review and meta-analysis. Expert Rev Gastroenterol Hepatol (2021) 15(11):1319–28. doi: 10.1080/17474124.2021.197053 34488515

[22] Liu M Xing J Xu K Yuan P Cui M Zhang C . Application of near-infrared fluorescence imaging with indocyanine green in totally laparoscopic distal gastrectomy. J Gastric Cancer (2020) 20(3):290–9. doi: 10.5230/jgc.2020.20.e25 PMC7521987 33024585

[23] Lu X Liu S Xia X Sun F Liu Z Wang J . The short-term and long-term outcomes of indocyanine green tracer-guided laparoscopic radical gastrectomy in patients with gastric cancer. World J Surg Oncol (2021) 19(1):271. doi: 10.1186/s12957-021-02385-1 34503530 PMC8431906

[24] Romanzi A Mancini R Ioni L Picconi T Pernazza G . ICG-NIR-guided lymph node dissection during robotic subtotal gastrectomy for gastric cancer. a single-centre experience. Int J Med Robot (2021) 17(2):e2213. doi: 10.1002/rcs.2213 33372409

[25] Park SH Berlth F Choi JH Park JH Suh YS Kong SH . Near-infrared fluorescence-guided surgery using indocyanine green facilitates secure infrapyloric lymph node dissection during laparoscopic distal gastrectomy. Surg Today (2020) 50(10):1187–96. doi: 10.1007/s00595-020-01993-w 32246228

[26] Lee S Song JH Choi S Cho M Kim YM Kim HI . Fluorescent lymphography during minimally invasive total gastrectomy for gastric cancer: an effective technique for splenic hilar lymph node dissection. Surg Endosc (2021) 36(5):2914-24. doi: 10.1007/s00464-021-08584-x 34109482

[27] Roh CK Choi S Seo WJ Cho M Son T Kim HI . Indocyanine green fluorescence lymphography during gastrectomy after initial endoscopic submucosal dissection for early gastric cancer. Br J Surg (2020) 107(6):712–9. doi: 10.1002/bjs.11438 32031248

[28] Ma S Zhang YM Dou LZ Liu H Ma FH Wang GQ . Efficacy and feasibility of indocyanine green for mapping lymph nodes in advanced gastric cancer patients undergoing laparoscopic distal gastrectomy. J Gastrointest Surg (2020) 24(10):2306–9. doi: 10.1007/s11605-020-04706-3 32607859

[29] Tian Y Lin Y Guo H Hu Y Li Y Fan L . Safety and efficacy of carbon nanoparticle suspension injection and indocyanine green tracer-guided lymph node dissection during robotic distal gastrectomy in patients with gastric cancer. Surg Endosc (2021) 36(5):3209-16. doi: 10.1007/s00464-021-08630-8 PMC9001219 34254184

[30] Moher D Shamseer L Clarke M Ghersi D Liberati A Petticrew M . Preferred reporting items for systematic review and meta-analysis protocols (PRISMA-p) 2015 statement. Syst Rev (2015) 4(1):1. doi: 10.1186/2046-4053-4-1 25554246 PMC4320440

[31] Yoshii A Plaut DA McGraw KA Anderson MJ Wellik KE . Analysis of the reporting of search strategies in cochrane systematic reviews. J Med Libr Assoc (2009) 97(1):21–9. doi: 10.3163/1536-5050.97.1.004 PMC2605027 19158999

[32] Stang A . Critical evaluation of the Newcastle-Ottawa scale for the assessment of the quality of nonrandomized studies in meta-analyses. Eur J Epidemiol (2010) 25(9):603–5. doi: 10.1007/s10654-010-9491-z 20652370

[33] Wan X Wang W Liu J Tong T . Estimating the sample mean and standard deviation from the sample size, median, range and/or interquartile range. BMC Med Res Methodol (2014) 14:135. doi: 10.1186/1471-2288-14-135 25524443 PMC4383202

[34] Kwon IG Son T Kim HI Hyung WJ . Fluorescent lymphography-guided lymphadenectomy during robotic radical gastrectomy for gastric cancer. JAMA Surg (2019) 154(2):150–8. doi: 10.1001/jamasurg.2018.4267 PMC6439673 30427990

[35] Cianchi F Indennitate G Paoli B Ortolani M Lami G Manetti N . The clinical value of fluorescent lymphography with indocyanine green during robotic surgery for gastric cancer: A matched cohort study. J Gastrointest Surg (2020) 24(10):2197–203. doi: 10.1007/s11605-019-04382-y 31485904

[36] Lan YT Huang KH Chen PH Liu CA Lo SS Wu CW . A pilot study of lymph node mapping with indocyanine green in robotic gastrectomy for gastric cancer. SAGE Open Med (2017) 5:2050312117727444. doi: 10.1177/2050312117727444 28856007 PMC5570112

[37] Qian Y Cai S . A safe and effective surgical navigation technique in laparoscopic radical gastrectomy: Indocyanine green-mediated near-infrared fluorescent imaging. Cancer Commun (Lond) (2020) 40(6):270–2. doi: 10.1002/cac2.12033 PMC7307247 32525596

[38] Jeremiasse B van den Bosch CH Wijnen MWHA Terwisscha van Scheltinga CEJ Fiocco MF van der Steeg AFW . Systematic review and meta-analysis concerning near-infrared imaging with fluorescent agents to identify the sentinel lymph node in oncology patients. Eur J Surg Oncol (2020) 46(11):2011–22. doi: 10.1016/j.ejso.2020.07.012 32826112

[39] Chen QY Zhong Q Li P Xie JW Liu ZY Huang XB . Comparison of submucosal and subserosal approaches toward optimized indocyanine green tracer-guided laparoscopic lymphadenectomy for patients with gastric cancer (FUGES-019): A randomized controlled trial. BMC Med (2021) 19(1):276. doi: 10.1186/s12916-021-02125-y 34702260 PMC8549272

[40] Vahrmeijer AL Hutteman M van der Vorst JR van de Velde CJ Frangioni JV . Image-guided cancer surgery using near-infrared fluorescence. Nat Rev Clin Oncol (2013) 10(9):507–18. doi: 10.1038/nrclinonc.2013.123 PMC3755013 23881033

[41] Kim DW Jeong B Shin IH Kang U Lee Y Park YS . Sentinel node navigation surgery using near-infrared indocyanine green fluorescence in early gastric cancer. Surg Endosc (2019) 33(4):1235–43. doi: 10.1007/s00464-018-6401-z 30167947

[42] Son T Hyung WJ Lee JH Kim YM Kim HI An JY . Clinical implication of an insufficient number of examined lymph nodes after curative resection for gastric cancer. Cancer (2012) 118(19):4687–93. doi: 10.1002/cncr.27426 22415925

[43] Mocellin S Nitti D . Lymphadenectomy extent and survival of patients with gastric carcinoma: A systematic review and meta-analysis of time-to-event data from randomized trials. Cancer Treat Rev (2015) 41(5):448–54. doi: 10.1016/j.ctrv.2015.03.003 25814393

[44] Kiyokawa T Fukagawa T . Recent trends from the results of clinical trials on gastric cancer surgery. Cancer Commun (Lond) (2019) 39(1):11. doi: 10.1186/s40880-019-0360-1 30917873 PMC6437915

[45] Hirche C Mohr Z Kneif S Doniga S Murawa D Strik M . Ultrastaging of colon cancer by sentinel node biopsy using fluorescence navigation with indocyanine green. Int J Colorectal Dis (2012) 27(3):319–24. doi: 10.1007/s00384-011-1306-5 21912878

[46] Noura S Ohue M Seki Y Tanaka K Motoori M Kishi K . Feasibility of a lateral region sentinel node biopsy of lower rectal cancer guided by indocyanine green using a near-infrared camera system. Ann Surg Oncol (2010) 17(1):144–51. doi: 10.1245/s10434-009-0711-2 19774415

[47] Andersen HS Bennedsen ALB Burgdorf SK Eriksen JR Eiholm S Toxværd A . *In vivo* and *ex vivo* sentinel node mapping does not identify the same lymph nodes in colon cancer. Int J Colorectal Dis (2017) 32(7):983–90. doi: 10.1007/s00384-017-2777-9 28210851

[48] Yano K Nimura H Mitsumori N Takahashi N Kashiwagi H Yanaga K . The efficiency of micrometastasis by sentinel node navigation surgery using indocyanine green and infrared ray laparoscopy system for gastric cancer. Gastric Cancer (2012) 15(3):287–91. doi: 10.1007/s10120-011-0105-6 22041868

[49] Villegas-Tovar E Jimenez-Lillo J Jimenez-Valerio V Diaz-Giron-Gidi A Faes-Petersen R Otero-Piñeiro A . Performance of indocyanine green for sentinel lymph node mapping and lymph node metastasis in colorectal cancer: A diagnostic test accuracy meta-analysis. Surg Endosc (2020) 34(3):1035–47. doi: 10.1007/s00464-019-07274-z 31754853

[50] Shao J Zheng X Feng L Lan T Ding D Cai Z . Targeting fluorescence imaging of RGD-modified indocyanine green micelles on gastric cancer. Front Bioeng Biotechnol (2020), 8:575365. doi: 10.3389/fbioe.2020.575365 33102459 PMC7546337

[51] Sitarz R Skierucha M Mielko J Offerhaus GJA Maciejewski R Polkowski WP . Gastric cancer: Epidemiology, prevention, classification, and treatment. Cancer Manag Res (2018), 10:239–248. doi: 10.2147/CMAR.S149619 PMC5808709 29445300

[52] GBD 2017 Stomach Cancer Collaborators . The global, regional, and national burden of stomach cancer in 195 countries, 1990-2017: A systematic analysis for the global burden of disease study 2017. Lancet Gastroenterol Hepatol (2020) 5(1):42–54. doi: 10.1016/S2468-1253(19)30328-0 31648970 PMC7033564

