# Supplementary material for: Safety and efficacy of indocyanine green near-infrared fluorescent imaging-guided lymph nodes dissection during radical gastrectomy for gastric cancer: A systematic review and meta-analysis
Source: Front Oncol. 2022 Aug 16;12:917541. doi: 10.3389/fonc.2022.917541 (PMC9425773; doi:10.3389/fonc.2022.917541)

**Pubmed**

**((stomach neoplasms[MeSH Terms]) OR ((((((((((((((((((Neoplasm, Stomach[Title/Abstract]) OR (Stomach Neoplasm[Title/Abstract])) OR (Neoplasms, Stomach[Title/Abstract])) OR (Gastric Neoplasms[Title/Abstract])) OR (Gastric Neoplasm[Title/Abstract])) OR (Neoplasm, Gastric[Title/Abstract])) OR (Neoplasms, Gastric[Title/Abstract])) OR (Cancer of Stomach[Title/Abstract])) OR (Stomach Cancers[Title/Abstract])) OR (Gastric Cancer[Title/Abstract])) OR (Cancer, Gastric[Title/Abstract])) OR (Cancers, Gastric[Title/Abstract])) OR (Gastric Cancers[Title/Abstract])) OR (Stomach Cancer[Title/Abstract])) OR (Cancer, Stomach[Title/Abstract])) OR (Cancers, Stomach[Title/Abstract])) OR (Cancer of the Stomach[Title/Abstract])) OR (Gastric Cancer, Familial Diffuse[Title/Abstract]))) AND ((Indocyanine Green[MeSH Terms]) OR ((((((((Green, Indocyanine[Title/Abstract]) ) OR (Wofaverdin[Title/Abstract])) OR (Vophaverdin[Title/Abstract])) OR (Ujoveridin[Title/Abstract])) OR (Vofaverdin[Title/Abstract])) OR (Cardio-Green[Title/Abstract])) OR (Cardio Green[Title/Abstract])) OR (Cardiogreen[Title/Abstract])))**

**(((**Procedure, Robotic Surgical**[Title/Abstract]) OR (**Procedures, Robotic Surgical**[Title/Abstract])) OR (**Robotic Surgical Procedure**[Title/Abstract])) OR (**Surgical Procedure, Robotic**[Title/Abstract])) OR (**Robot Surgery**[Title/Abstract])) OR (**Robot Surgeries**[Title/Abstract])) OR (**Surgery, Robot**[Title/Abstract])) OR (**Robot-Assisted Surgery**[Title/Abstract])) OR (**Robot Assisted Surgery**[Title/Abstract])) OR (**Robot-Assisted Surgeries**[Title/Abstract])) OR (**Surgery, Robot-Assisted**[Title/Abstract])) OR (**Robot-Enhanced Procedures**[Title/Abstract])) OR (**Procedure, Robot-Enhanced**[Title/Abstract])) OR (**Robot Enhanced Procedures**[Title/Abstract])) OR (**Robot-Enhanced Procedure**[Title/Abstract])) OR (**Surgical Procedures, Robotic**[Title/Abstract])) OR (**Robotic-Assisted Surgery**[Title/Abstract])) OR (**Robotic Assisted Surgery**[Title/Abstract])) OR(**Robotic-Assisted Surgeries**[Title/Abstract])) OR(**Surgery, Robotic-Assisted**[Title/Abstract])) OR(**Robot-Enhanced Surgery**[Title/Abstract])) OR(**Robot Enhanced Surgery**[Title/Abstract])) OR(**Robot-Enhanced Surgeries**[Title/Abstract])) OR(**Surgery, Robot-Enhanced**[Title/Abstract])))**

****
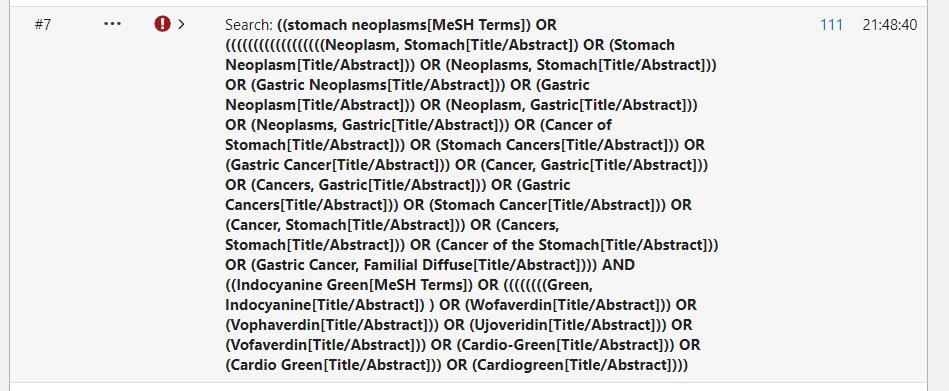
****

****EMBESE****

**#1 'stomach tumor'/exp**

**#2(Neoplasm, Stomach):ab,ti,kw OR (Stomach Neoplasm):ab,ti,kw OR (Neoplasms, Stomach):ab,ti,kw OR (Gastric Neoplasms):ab,ti,kw OR (Gastric Neoplasm):ab,ti,kw OR (Neoplasm, Gastric):ab,ti,kw OR (Neoplasms, Gastric):ab,ti,kw OR (Cancer of Stomach):ab,ti,kw OR (Stomach Cancers):ab,ti,kw OR (Gastric Cancer):ab,ti,kw OR (Cancer, Gastric):ab,ti,kw OR (Cancers, Gastric):ab,ti,kw OR (Gastric Cancers):ab,ti,kw OR (Stomach Cancer):ab,ti,kw OR (Cancer, Stomach):ab,ti,kw OR (Cancers, Stomach):ab,ti,kw OR (Cancer of the Stomach):ab,ti,kw OR (Gastric Cancer, Familial Diffuse):ab,ti,kw**

**#3 'Indocyanine green'/exp**

**#4 (Green, Indocyanine):ab,ti,kw OR (Wofaverdin):ab,ti,kw OR (Vophaverdin):ab,ti,kw OR (Ujoveridin):ab,ti,kw OR (Vofaverdin):ab,ti,kw OR (Cardio-Green):ab,ti,kw OR (Cardio Green):ab,ti,kw OR (Cardiogreen):ab,ti,kw**

**#5 #1 OR #2**

**#6 #3 OR #4**

**#7 #3 AND #4**

****
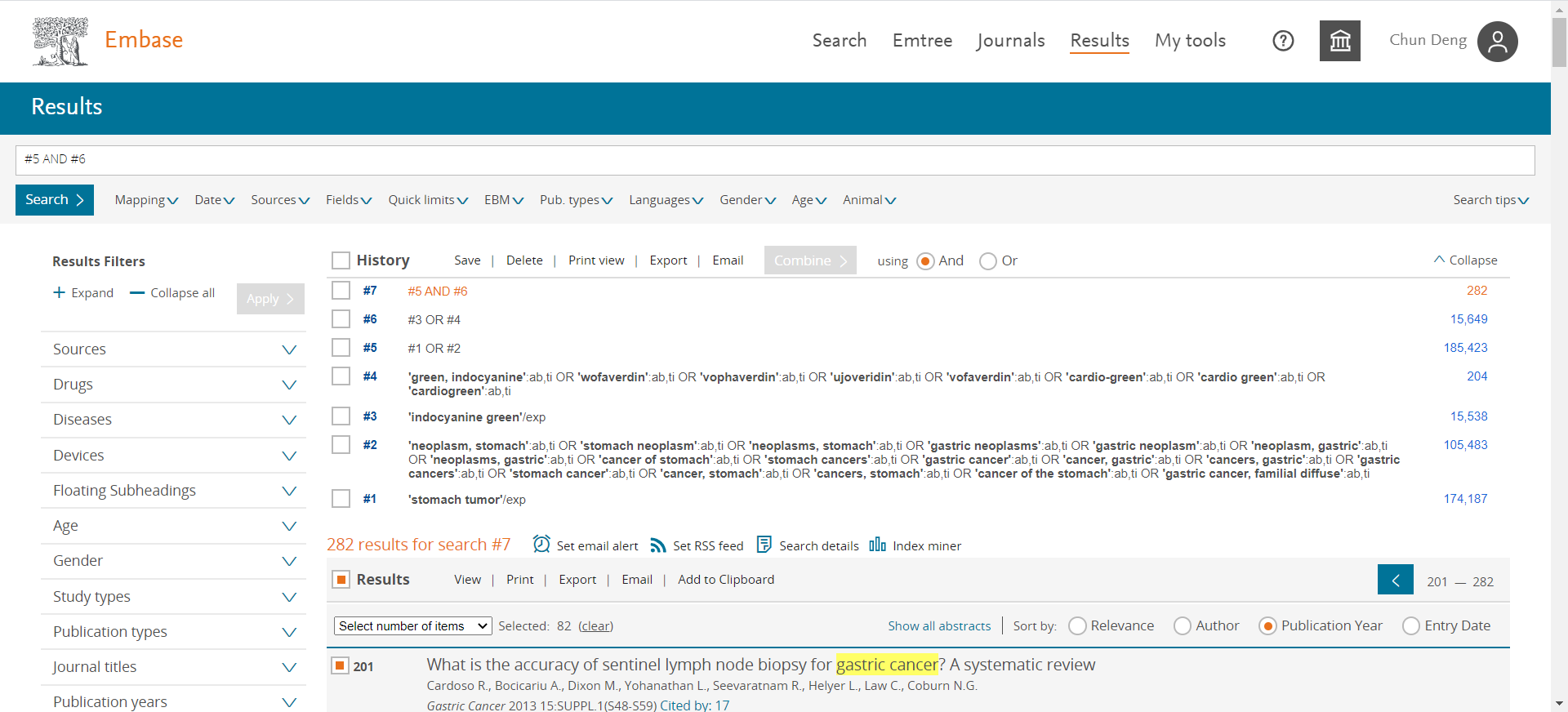
****

**Web of Science**

#1 TS=(Indocyanine Green OR Green, Indocyanine OR Wofaverdin OR Vophaverdin OR Ujoveridin OR Vofaverdin OR Cardio-Green OR Cardio Green OR Cardiogreen)

#2 TS=(stomach neoplasms OR Neoplasm, Stomach OR Stomach Neoplasm OR Neoplasms, Stomach OR Gastric Neoplasms OR Gastric Neoplasm OR Neoplasm, Gastric OR Neoplasms, Gastric OR Stomach Cancers OR Cancer of Stomach OR Gastric Cancer OR Cancer, Gastric OR Cancers, Gastric OR Gastric Cancers OR Stomach Cancer OR Cancers, Stomach OR Cancer, Stomach OR Cancer of the Stomach OR Gastric Cancer, Familial Diffuse)

#3 #1 AND #2


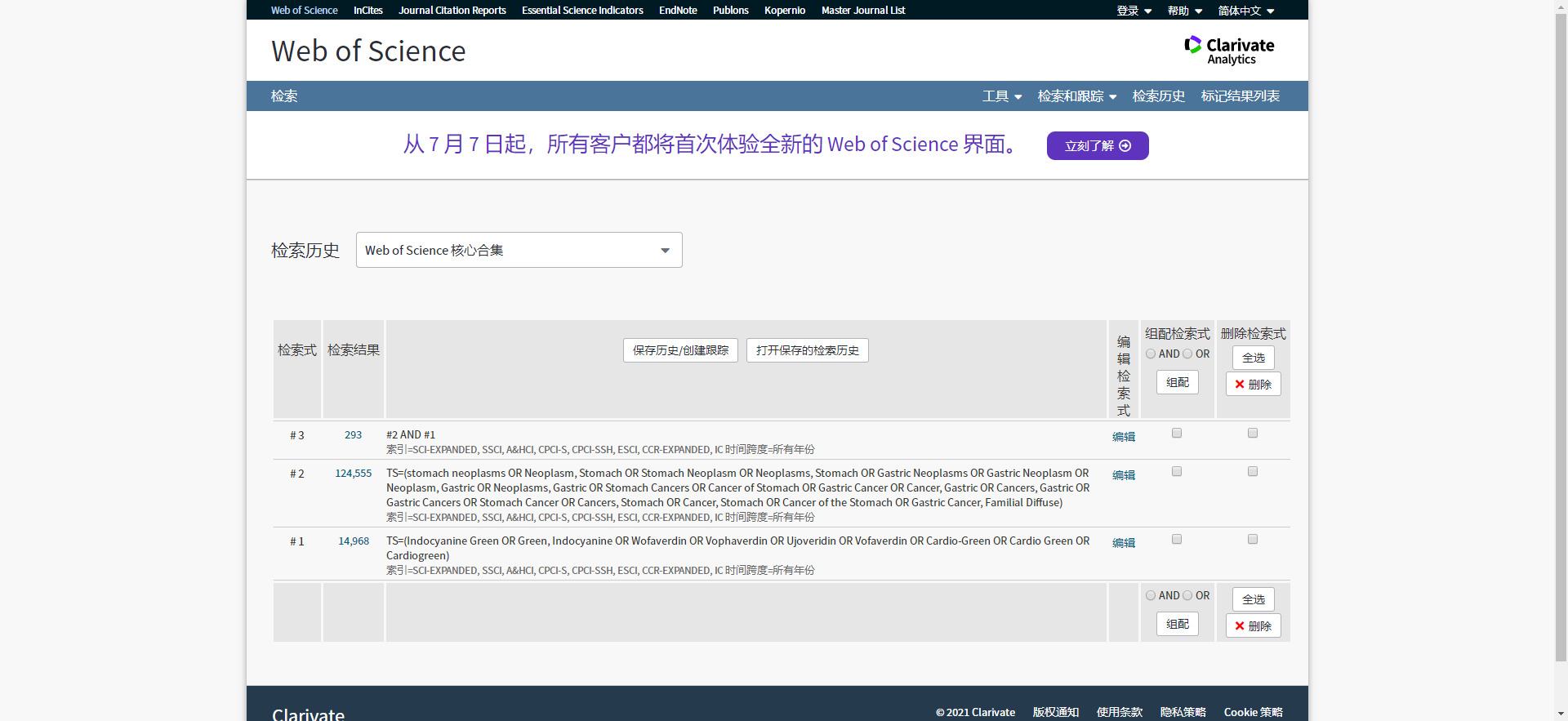


**Cochrane Library**

#1 MeSH descriptor:[**stomach neoplasms**] explode all trees

#2 (Neoplasm, Stomach):ab,ti,kw OR (Stomach Neoplasm):ab,ti,kw OR (Neoplasms, Stomach):ab,ti,kw OR (Gastric Neoplasms):ab,ti,kw OR (Gastric Neoplasm):ab,ti,kw OR (Neoplasm, Gastric):ab,ti,kw OR (Neoplasms, Gastric):ab,ti,kw OR (Cancer of Stomach):ab,ti,kw OR (Stomach Cancers):ab,ti,kw OR (Gastric Cancer):ab,ti,kw OR (Cancer, Gastric):ab,ti,kw OR (Cancers, Gastric):ab,ti,kw OR (Gastric Cancers):ab,ti,kw OR (Stomach Cancer):ab,ti,kw OR (Cancer, Stomach):ab,ti,kw OR (Cancers, Stomach):ab,ti,kw OR (Cancer of the Stomach):ab,ti,kw OR (Gastric Cancer, Familial Diffuse):ab,ti,kw

#3 #1 OR #2

#4 MeSH descriptor:[**Indocyanine green**] explode all trees

#5 (Green, Indocyanine):ab,ti,kw OR (Wofaverdin):ab,ti,kw OR (Vophaverdin):ab,ti,kw OR (Ujoveridin):ab,ti,kw OR (Vofaverdin):ab,ti,kw OR (Cardio-Green):ab,ti,kw OR (Cardio Green):ab,ti,kw OR (Cardiogreen):ab,ti,kw

#6 #4 OR #5

#7 #3 AND #6


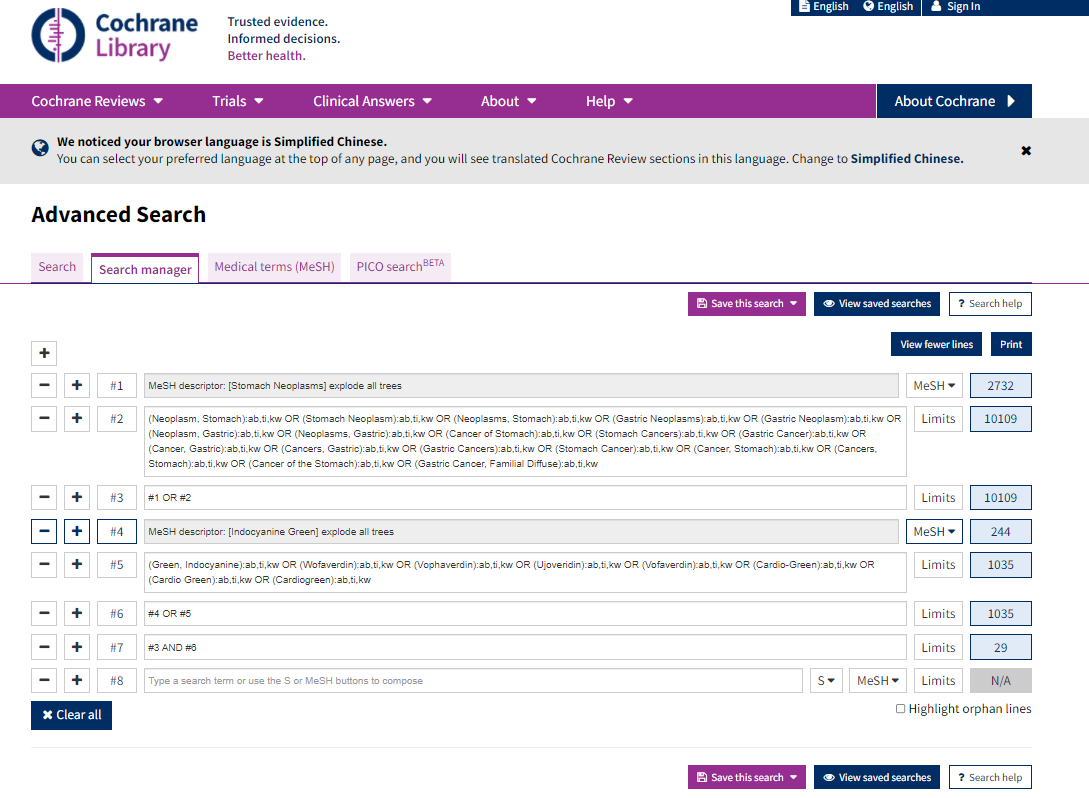

Supplement: Supplementary file 1 [file DataSheet_1.doc]
