# Supplementary material for: Safety and efficacy of indocyanine green near-infrared fluorescent imaging-guided lymph nodes dissection during radical gastrectomy for gastric cancer: A systematic review and meta-analysis
Source: Front Oncol. 2022 Aug 16;12:917541. doi: 10.3389/fonc.2022.917541 (PMC9425773; doi:10.3389/fonc.2022.917541)
Supplement: Supplementary file 2 [file DataSheet_2.doc]

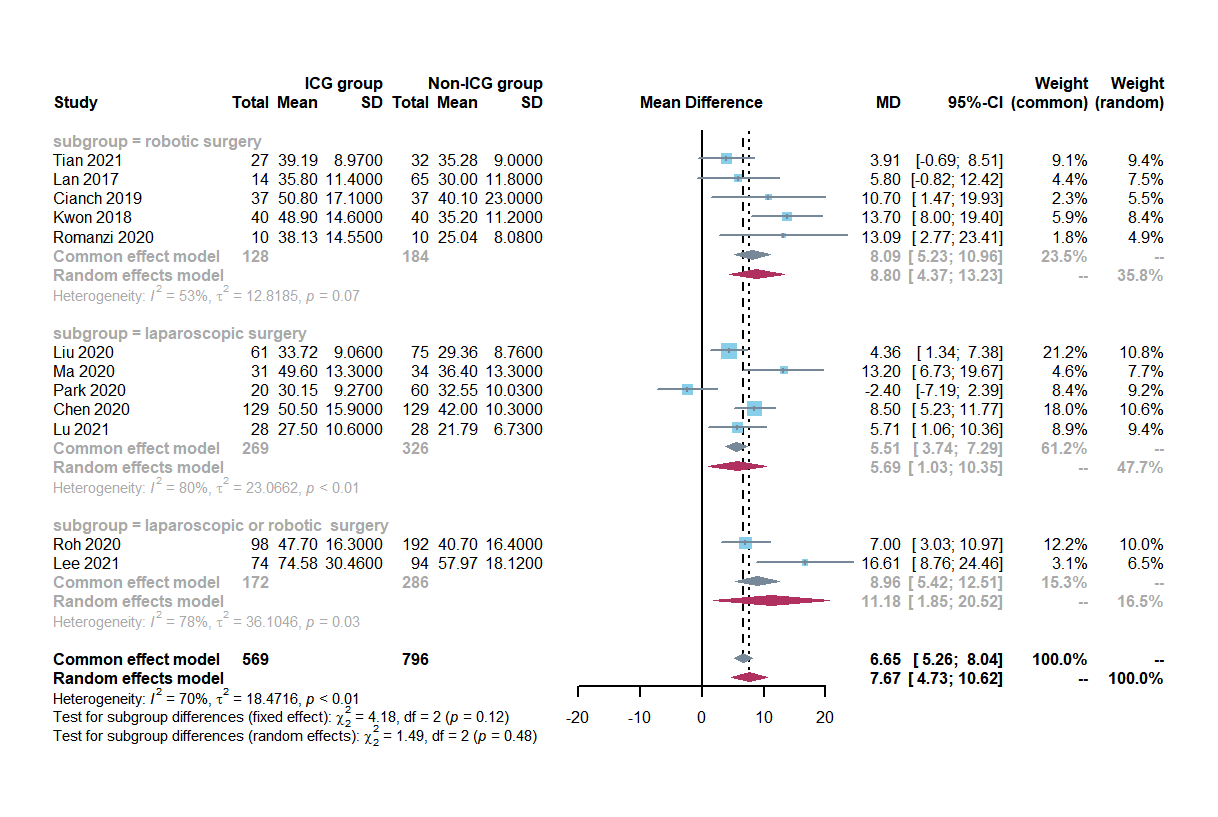


**Fig.1** Forest plots of subgroup analysis based on “operation type”.


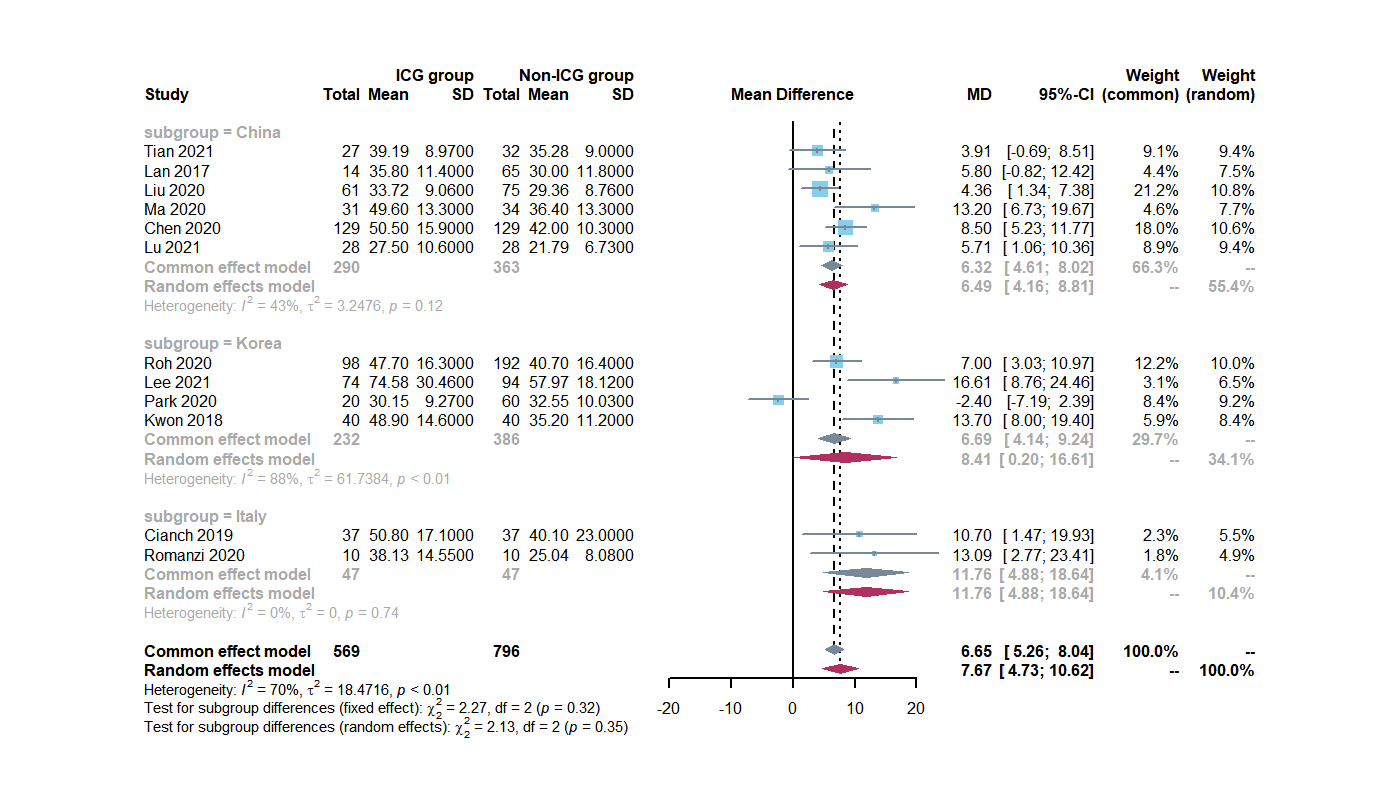


**Fig.2** Forest plots of subgroup analysis based on “nation”.


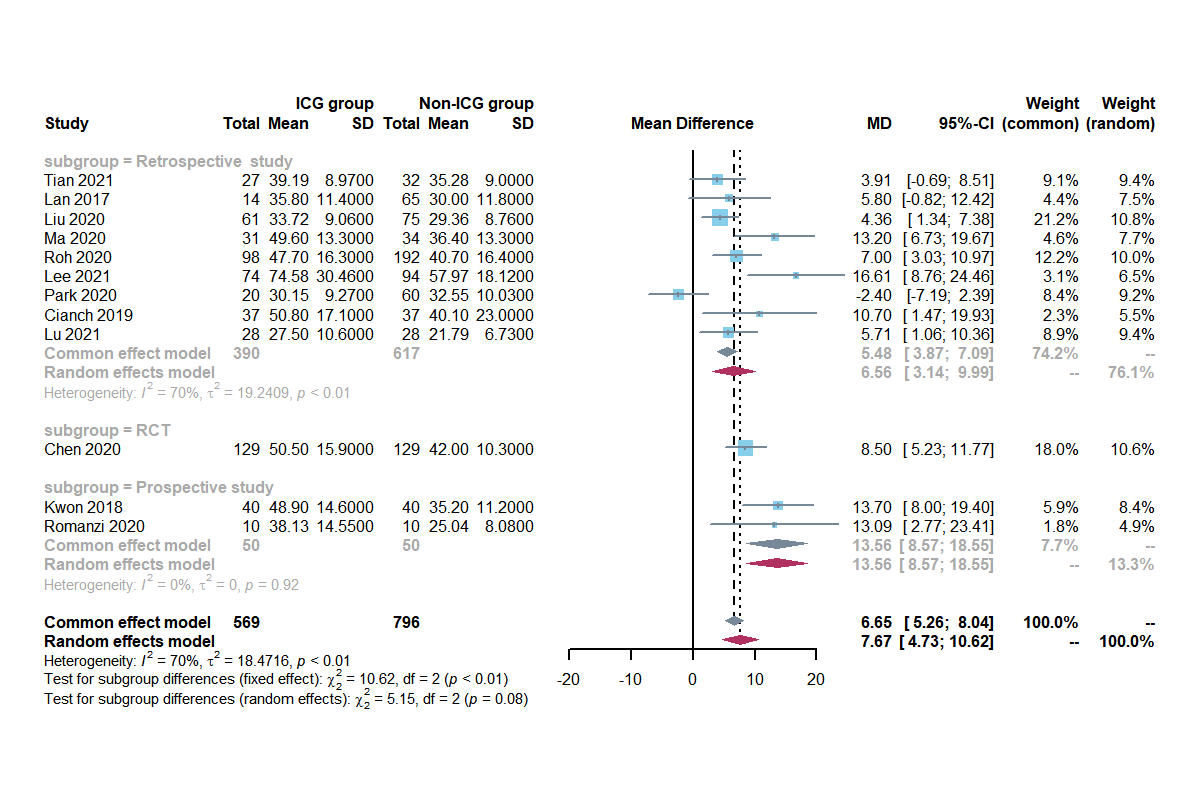


**Fig.3** Forest plots of subgroup analysis based on “study design”.
