# Supplementary figures and images for: Safety and efficacy of indocyanine green near-infrared fluorescent imaging-guided lymph nodes dissection during radical gastrectomy for gastric cancer: A systematic review and meta-analysis
Source: Front Oncol. 2022 Aug 16;12:917541. doi: 10.3389/fonc.2022.917541 (PMC9425773; doi:10.3389/fonc.2022.917541)

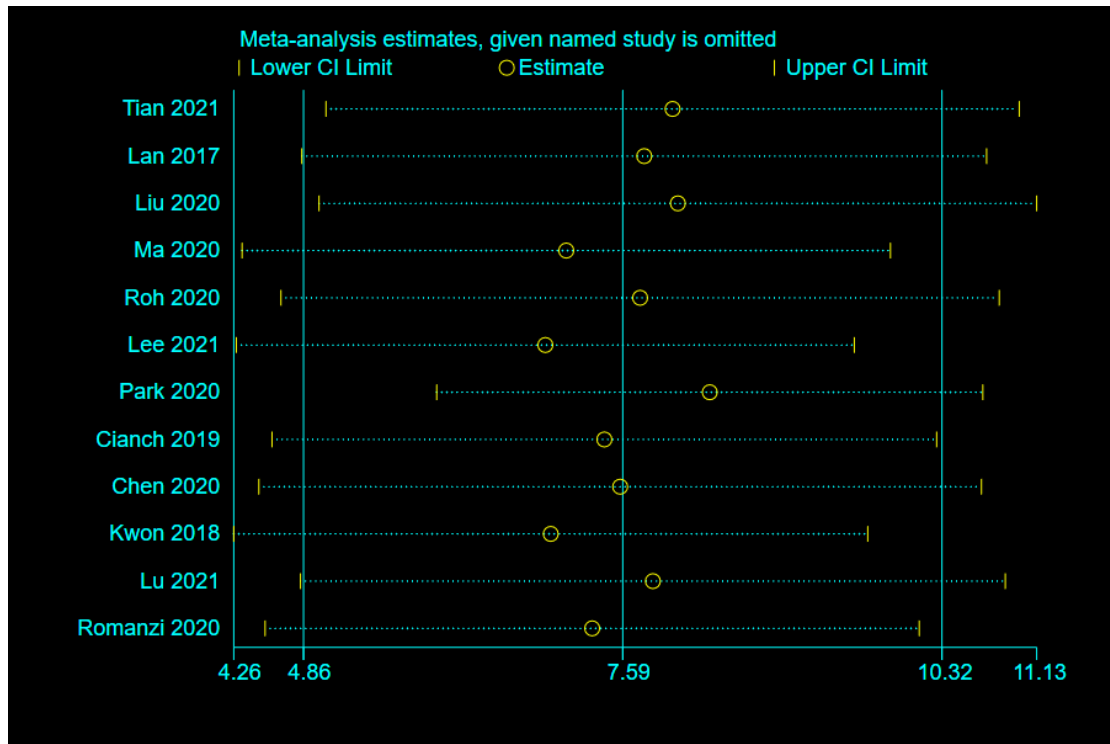

**Fig1** Figure of Sensitivity analysis

Supplement: Supplementary file 3 [file Image_1.pdf]
